# Carvacrol, a Plant Metabolite Targeting Viral Protease (M^pro^) and ACE2 in Host Cells Can Be a Possible Candidate for COVID-19

**DOI:** 10.3389/fpls.2020.601335

**Published:** 2021-02-16

**Authors:** Hayate Javed, Mohamed Fizur Nagoor Meeran, Niraj Kumar Jha, Shreesh Ojha

**Affiliations:** ^1^Department of Anatomy, College of Medicine and Health Sciences, United Arab Emirates University, Al Ain, United Arab Emirates; ^2^Department of Pharmacology and Therapeutics, College of Medicine and Health Sciences, United Arab Emirates University, Al Ain, United Arab Emirates; ^3^Department of Biotechnology, School of Engineering and Technology (SET), Sharda University, Knowledge Park III, Greater Noida, India

**Keywords:** carvacrol, terpenoid, M^pro^ binding, ACE2 receptor, SARS-CoV-2

## Abstract

The recent outbreak of severe acute respiratory syndrome coronavirus 2 (SARS-CoV-2) started in December 2019, resulting in the coronavirus disease-19 (COVID-19) pandemic. Coronaviruses are solely accountable for rising mortality and socioeconomic saddles. Presently, there are few repurposed drugs such as remdesivir or favipiravir approved for the treatment of COVID-19, although vaccines and plasma therapy is also subject to emergency approval. However, some potential natural treatments and cures have also been proposed. Molecules of natural origin showed therapeutic importance such as antiviral, anti-inflammatory, and antioxidant activity, and could be useful drug candidates for treating COVID-19. In recent years, essential oils have shown promising therapeutic effects against many viral diseases. Carvacrol is one of the monoterpene phenol with abundant presence in essential oils of many aromatic plants, including thyme and oregano. It is being used as food flavoring, additive, and preservatives. Carvacrol is also used as a fragrance in cosmetic products. A number of research studies have shown biological actions of carvacrol with its therapeutic potential is of clinical significance. The *in vitro* and *in vivo* studies have shown multiple pharmacological properties such as anticancer, anti-fungal, anti-bacterial, anti-oxidant, anti-inflammatory, vasorelaxant, hepatoprotective, and spasmolytic. This review highlights the various biological and pharmacological properties of carvacrol within the scope of COVID-19.

## Introduction

The novel virus strain, SARS-CoV-2 is responsible for the recent outbreak of respiratory infectious disease known as “coronavirus disease 19” (COVID 19) (Boopathi et al., 2020; Hemida and Ba Abduallah, 2020). Globally, the health and healthcare systems are under serious threat due to this major outbreak (Lu et al., 2020; Zhu et al., 2020). The pandemic has affected millions of individuals because of compulsory quarantine and isolation, and also devastated the healthcare facilities. The pandemic have a severe impact on the world economy and will continue to impact if the spread of this novel virus is not stopped or a valuable treatment is not discovered. Coronaviruses are transmitted to humans through respiratory droplets and are single-stranded positive-sense RNA viruses. Patients infected with SARS-CoV-2 display many symptoms including fever, dry cough, diarrhea, loss of smell, and complications mainly beginning with acute respiratory distress following a rapid and robust rise in the levels of proinflammatory cytokines (Das et al., 2020; Jean et al., 2020; Zhang et al., 2020). The morphology of the coronavirus consists of transmembrane spike glycoproteins (S protein), which project outside from the surface of the virus (Tortorici and Veesler, 2019). SARS-CoV and SARS-CoV-2 both display structural morphology in their S proteins and preserved ectodomains. In light of previous studies, the binding inhibition of SARS-CoV to its host cell receptor angiotensin-converting enzyme-2 (ACE2) appears most relevant, as the cellular entry of SARS-CoV-2 also requires ACE2 (Hoffmann et al., 2020; Walls et al., 2020). The epithelial cells of the respiratory tract express ACE2, an exopeptidase, which may provide a potentially viable pharmacological approach to control the cell entry of SARS-CoV-2. Infections with SARS-CoV-2 may affect the gastrointestinal tract, central nervous system, kidney, liver, and heart (Tang et al., 2020). In comparison to SARS-CoV or MERS-CoV, SARS-CoV-2 is highly infectious and communicable (Baez-Santos et al., 2015).

In studies on the replication and infection processes of SARS-CoV-2, numerous mechanisms have been suggested that could be targeted on a pharmacological basis for prevention and treatment. The viral S protein is required by the mast cells of macrophages, pneumocytes, and pulmonary cells in infections. A diverse range of viruses require host cell proteases to activate the S glycoprotein for cellular entry (Bertram et al., 2012; Yamaya et al., 2015; Zhou et al., 2015). The membrane fusion and host cell entry require cleavage and activation of the spike protein (S protein) of SARS-CoV and are mediated by transmembrane protease/serine subfamily member 2 (TMPRSS2), an airway and alveolar cell serine protease (Matsuyama et al., 2010; Glowacka et al., 2011; Shulla et al., 2011). SARS-CoV-2 also involves TMPRSS2 for the priming of spike protein (S) driven cellular entry (Hoffmann et al., 2020).

A clinically established and commercially available, serine protease inhibitor, camostat mesylate, has partially inhibited the infection by HCoV-NL63 and SARS-CoV in HeLa cell lines, which express TMPRSS2 and ACE2 (Kawase et al., 2012). Camostat mesylate has also significantly inhibited the TMPRSS2 in human lung Calu-3 cells and lowered the infection with SARS-CoV-2 (Hoffmann et al., 2020). The viral host cell entry could be blocked by agents that inhibit TMPRSS2. Upon host cell entry, viral RNA (single-stranded) starts the replication process and subsequent translation of polyproteins which are eventually broken into full effecter proteins by the action of viral proteases (Baez-Santos et al., 2015). A viral infection is initiated by the interaction of S protein with ACE2 on the host cell cytoplasmic membrane.

Therapeutic strategies that disrupt the interaction of S protein with ACE2 could be of therapeutic importance. Recently, it was shown that the S protein of SARS-CoV-2 binding affinity to ACE2 is 10-20-times greater in comparison to the S protein of SARS-CoV indicating that the SARS-CoV-2 contagiousness is much higher than SARS-CoV (Tang et al., 2020). Recently, an *in vivo* study showed that multiple drugs including ritonavir/lopinavir and remdesivir targeted the MERS-CoV (Sheahan et al., 2020), and inhibited the RdRp of Ebola virus as well as the proteases of SARS-CoV-2 in humans. Moreover, these drugs are also recognized as potential drug candidates against SARS-CoV-2. The therapeutic efficacy of these drugs is now under investigation in two international clinical trials (SOLIDARITY Trial and DisCoVeRy Trial).

Numerous efforts are currently ongoing to accelerate the discovery and development of effective preventive and therapeutic candidate drugs against SARS-CoV-2 infections (Altay et al., 2020). In the past few months, since the emergence of COVID-19, several compounds have provided promising alternatives such as chloroquine, hydroxychloroquine, ritonavir, remdesivir, tocilizumab, interferon-β, ivermectin, lopinavir, ribavirin, and azithromycin (Wu et al., 2020). The repurposing of drugs has to date mainly concentrated on the pharmacological properties including antivirals, antibiotics, anti-inflammatory, and immunomodulators (Fan et al., 2020). The use of the above-mentioned drugs in COVID-19 patients is mostly empirical due to its lack of randomized controlled trials to demonstrate the efficacy and safety of these treatments. Taking into account COVID-19 related mortality, effective medications are required to improve the prognosis of patients and to curb the spread of the virus (Fan et al., 2020). Given the pharmacological perspective, all these drugs have the potential to either block the virus from entering host cells or prevent viral replication and/or attenuate the exacerbation of the host’s immune response (Fan et al., 2020).

The pathogenesis and complications caused by SARS-CoV-2 are primarily based on an immune-inflammatory cascade. Taking this into account, therapeutic approaches should be focused on this cascade by attenuating inflammation and immune modulation (Allegra et al., 2020; Song et al., 2020). Numerous researches are currently in progress across different institutions around the world to identify novel drug candidates as well as vaccine for COVID-19. Learning from the discovery of Tamiflu^®^, phytochemicals, and natural products with antiviral, anti-inflammatory, and immunomodulatory properties should be investigated for the prevention and cure of SARS-CoV-2 infections. Among all these therapeutic approaches, natural products, mainly essential oils (EOs) have drawn much interest because of their robust use as anti-inflammatory, immunomodulator, and antioxidant as well as a source of novel antimicrobial, anti-inflammatory, and immunomodulator agents (Allegra et al., 2014; Sadgrove and Jones, 2019). EOs are well recognized for their strong antiviral, anti-inflammatory, and immunomodulatory activities (de Lavor et al., 2018; Asif et al., 2020; Gandhi et al., 2020). EO displays numerous beneficial effects in different diseases and produces systemic effects, consequently, it has been proposed as a possible candidate for evaluation in prevention and treatment of COVID-19 (Agatonovic-Kustrin et al., 2019).

In viral infections, EO may have an important role as therapeutics in ameliorating the redox immune-inflammatory cascade by interfering with the pathways related to inflammatory processes in allergic and infectious airways. EO has shown time-tested safety and efficacy and because of that, it has been used in traditional medicine and food for a long time (Dosoky and Setzer, 2018). EOs are predominantly found in aromatic plants and are a complex mixture of lipophilic as well as volatile terpene compounds. They have been consumed via diets and recognized for potent antioxidant, anti-inflammatory, immunomodulatory, and antimicrobial properties (Aziz et al., 2018). Numerous experimental studies along with some clinical trials showed that EOs could be important therapeutic agents for immune system-related diseases (de Lavor et al., 2018; Gandhi et al., 2020). EOs supplementation along with other compounds are also very well recognized for their activity against bacteria and viruses that lead to respiratory infections. These have reported safe, and synergistic with potent antihistamine, and antioxidant properties (Boskabady et al., 2003; Leigh-de Rapper and van Vuuren, 2020).

Among many active principles of EOs, one of the compounds, carvacrol, received special attention due to recent reports of its specific binding with M^pro^, a protease enzyme in the viral genome belonging to non-structural proteins showing a significant effect in the replication and maturation of SARS-CoV-2 (Kumar et al., 2020). In another recent study, carvacrol, a bioactive molecule in the EO of *Ammoides verticillata Briq*. was reported to inhibit ACE2 activity and suggested that it may block the host cell entry of SARS-CoV-2 (Abdelli et al., 2020). Both these studies demonstrate the potential of carvacrol on virus machinery as well as virus entry and the replication in the host cells. Moreover, Kulkarni et al. (2020) have reported that various monoterpenoid phenols including carvacrol have the potential to inhibit the binding of viral spike (S) glycoprotein to the host cell. Carvacrol has been docked against the S1 receptor binding domain of the spike protein, which is the key target for novel antiviral drugs, to ascertain their inhibitory effects based on their binding affinities.

Carvacrol (C_1__0_H_1__4_O), a monoterpenoid of the phenolic group [2-methyl-5-(1-methylethyl) phenol], found in an EO of ajwain (*Carum copticum* (L.) Benth. and Hook. f. ex C.B. Clarke), oregano (*Origanum vulgare* L.), Shirazi thyme (*Zataria multiflora* Boiss.), thyme (*Thymus vulgaris* L.), black cumin (*Nigella sativa* L.), wild bergamot [*Citrus aurantium bigaradia* (Loisel.) Brandis], pepperwort (*Lepidium flavum* Torr.), and other plants (Butt and Sultan, 2010; Tang et al., 2011; Fachini-Queiroz et al., 2012; Boskabady et al., 2014; Khazdair et al., 2018).

The biosynthetic pathway of carvacrol has been elucidated recently. Carvacrol is biosynthesized from isopentenyl diphosphate (IDP) and dimethylallyl diphosphate (DMADP), which are derived from the methylerythritol phosphate (MEP) pathway located in plastids (Rohmer et al., 1993; Dudareva et al., 2005). In the MEP pathway, which is involved in the biosynthesis of carvacrol, 1-deoxy-D-xylulose-5-phosphate (DXP) is irreversibly converted into 2-C-methyl-D-erythritol-4-phosphate (MEP) by a 1-deoxy-D-xylulose-5-phosphate reductoisomerase enzyme (DXR). This step has been described as the first committed step in the MEP pathway (Takahashi et al., 1998). Geranyl diphosphate synthase (GDS) is a key enzyme in this biosynthetic pathway, which catalyzes the head-to-tail condensation of IDP and DMADP to geranyl diphosphate (GDP) as the universal precursor of monoterpenes (Lichtenthaler, 1999). Subsequently, γ-terpinene synthase, which is a member of the monoterpene synthase family produces γ-terpinene through the cyclization of GDP. Furthermore, enzymes such as CYP71D180 and CYP71D181 belonging to the cytochrome P450 (CYP) monooxygenases, are also involved in further modification of γ-terpinene backbone to yield carvacrol (Crocoll et al., 2010).

**FIGURE 1:**
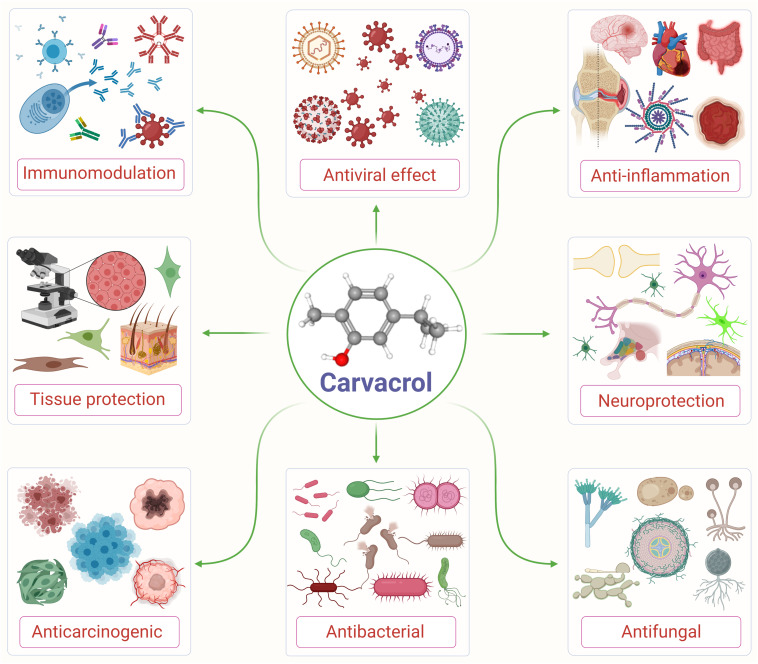
An illustrative flow diagram showing the various pharmacological properties of carvacrol and its chemical structure (center). Adopted and modified from Suntres et al. (2015).

Carvacrol is used as a microbicidal agent, and fragrance ingredient in cosmetic formulations (Andersen, 2006). Carvacrol is generally considered safe for consumption. It has been approved by the Federal Drug Administration for its use in food and is included by the Council of Europe in the list of chemical flavorings that can be found in alcoholic beverages, baked goods, chewing gum, condiment relish, frozen dairy, gelatin pudding, non-alcoholic beverages, and soft candy (Ultee et al., 2002; De Vincenzi et al., 2004). The structure and various pharmacological properties of carvacrol are represented in Figure 1.

**FIGURE 2:**
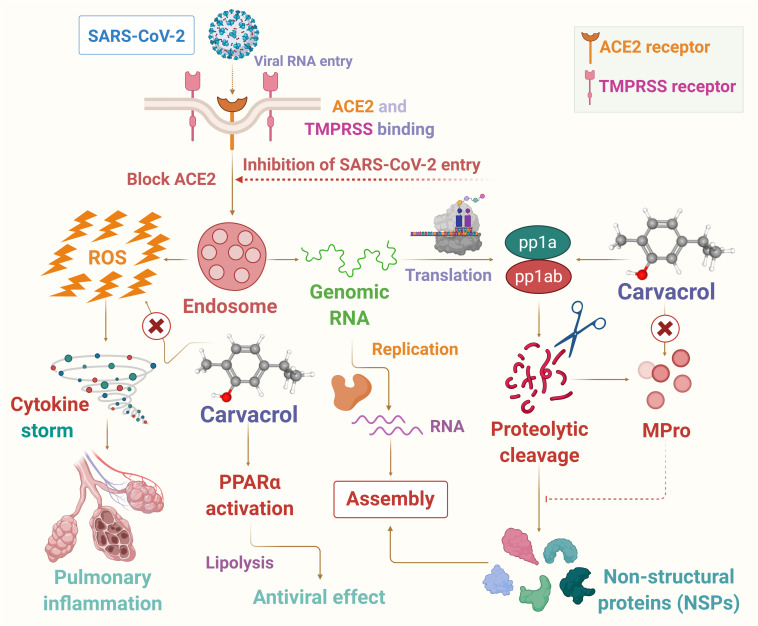
The possible mechanism of carvacrol on infection, immunity and inflammation in context to COVID-19.

In this article, it has been hypothesized that carvacrol could be a candidate for use as a preventive agent or therapeutic adjuvant in SARS-CoV-2 endowed with potent anti-inflammatory, antiviral, and immunomodulating properties to reduce the harshness and progression of the disease. In the present hypothesis the potential pharmacological and molecular mechanisms targeting oxidative stress, inflammation, immune system, and viremia or infectivity has been presented based on available literature that may provide logical speculation of its use in COVID-19. The proposed possible mechanisms of carvacrol on infection, immunity, and inflammation are presented in Figure 2.

## Carvacrol as an Immunomodulator Agent

The first line of defense is the immune system of the body, which plays an important role in all types of infections including SARS-CoV-2. Immuno-modulation is a process that targets and augments immune response to prevent infections in immuno-deficiencies. In the case of allergies or autoimmune diseases immuno-modulation aims to inhibit the immune system, where the target is to lessen the immune system. At the initial stage of SARS-CoV-2 infection, M1 phenotypic macrophages show up and release ROS, IL-8, IL-6, nitric oxide, MCP-1, IL-1, TNF-α, and CXCL-10, which modulate the host defense against the virus, however, at the same time, it also enhances lung injury. Inflammation is primarily initiated by TNF-α. Moreover, TNF-α is also a key player in cell death mediator, differentiation, and immune modulation. Anti-inflammatory macrophages (M2) are activated once the pathogenic agent is eliminated and eventually control the process of healing and restore the lung tissue. In addition, the innate immune system is significantly controlled by natural killer (NK) cells. At the time of infection, an innate immune response is initiated by eliminating the virally infected cells. Carvacrol administration to lipopolysaccharide (LPS) treated RAW 264.7 macrophages showed significant inhibition of LPS induced release of ROS, TNF-α, IL-1β, and NO. Moreover, carvacrol inhibited the nuclear translocation of the NF-κB (p65) subunit from the cytoplasm (Somensi et al., 2019).

Additionally, the virally infected cells are eliminated by the NK cells. Carvacrol treatment has been shown to attenuate the levels of pro- and anti- inflammatory cytokines in an animal model of experimental autoimmune encephalomyelitis. Carvacrol could be considered as a booster of autoimmunity as it ameliorated the levels of numerous cytokines such as IL-17, IFN-γ, TGF-β, IL-6, IL-10, and IL-4 (Mahmoodi et al., 2019). Carvacrol containing plant Zataria *multiflora* Boiss. also showed an immunomodulatory effect by augmenting the levels of IFN-γ and FOXP3 as well as IL-4, TGF-β, and IL-17 and could be useful in correcting immune dysregulation (Khazdair et al., 2018). In addition, carvacrol also showed strong anti-inflammatory and antioxidant properties, which could be beneficial in the cure of diseases associated with enhanced oxidative stress along with inflammatory and altered immune status (Khazdair et al., 2018). Adaptive immune responses (Giamarellos-Bourboulis et al., 2020; Kuri-Cervantes et al., 2020; Liao et al., 2020), mainly of T cells (Giamarellos-Bourboulis et al., 2020; Mathew et al., 2020), showed a significant role in the infection of SARS-CoV-2, similar to the infection of respiratory diseases. Carvacrol has been shown to reduce the transcription factors of T_H_ cell-related cytokines in ovalbumin immunized mice, thus suppressing the antigen-specific immune response, this indicates the potential of carvacrol to ameliorate the critical immune responses ascribed to the over activation of T-cells (Gholijani and Amirghofran, 2016).

## Carvacrol as an Anti-Inflammatory Agent

The inflammatory process is tackled through innate immune response and is consequently responsible for the release of inflammatory mediators (cytokines, ROS etc.) by the various cells of the immune system (neutrophils, macrophages, lymphocytes) that activates the adaptive immunity but enhances inflammation. T helper cells in turn result in the activation of various other types of cells (monocytes, B cells, etc.) by releasing cytokines, such as TNF-α, IL-1β, and IL-2, which institute the inflammatory cascade (Bradley, 2008).

Numerous studies have shown that the anti-inflammatory activity of carvacrol, derivatives of carvacrol, and plants containing a high amount of carvacrol in LPS-induced macrophages or monocytes or eosinophils, inhibit proinflammatory cytokines, inflammatory mediators, iNOS and COX-2, the production of NO and prostaglandin E2, and CD18 frequency on human lymphocytes (Lima Mda et al., 2013; Damasceno et al., 2014; Gholijani and Amirghofran, 2016; Li et al., 2018; Somensi et al., 2019). The hepatoprotective effect of carvacrol was observed in D-galactosamine-induced hepatotoxicity in rats. Carvacrol has been found to reduce CYP2E1 and enhance PPAR-α expression in alleviating liver damage (Aristatile et al., 2014). Carvacrol was also found as a COX-2 suppressor and PPAR-γ activator. Carvacrol has been shown to suppress the LPS induced mRNA and protein expression of COX-2 in human macrophage-like U937 cells, indicating that carvacrol ameliorates the expression of COX-2 via its agonistic effect on PPAR-γ (Hotta et al., 2010).

The role of activating PPAR-α and lipolysis has been shown to reduce the hepatitis C virus genotype-associated lipid metabolic disorder in liver diseases (Patra et al., 2019). PPAR-α activation has also been shown to beneficially influence inflammatory alveolar epithelial cells and suggest the potential usefulness of PPAR-α in acute respiratory distress syndrome (Hecker et al., 2015). Carvacrol a natural dietary molecule has no adverse effects and could be beneficial for synthetic PPAR-α. Carvacrol has been also found to activate PPAR-α and reduce the expression of mRNA and the protein of inflammatory mediator COX-2 induced by LPS (Hotta et al., 2010). Overall, carvacrol’s role as a PPAR-α agonist has also shown promising effects in regulating lipid metabolism in addition to its regulatory role on the immune system, cell proliferation, and differentiation, atherosclerosis vascular homeostasis, and inflammation (Lee et al., 2015; Gholijani and Amirghofran, 2016; Li et al., 2018). Moreover, *Nigella sativa* L., commonly known as black cumin, and its EO component carvacrol have shown strong pharmacological properties against dyslipidemia and respiratory disorders.

Carvacrol may also maintain the immune system balance through its role in immunomodulation (Butt and Sultan, 2010). Hence, carvacrol could be beneficial for limiting the orchestrated immune-inflammatory cascade in COVID-19.

### Carvacrol as an Antiviral Agent

EOs and extracts of plant origin have been investigated for various pharmacological activities including anti-viral activities (Oltmans et al., 1986). Carvacrol was one of the compounds of the EOs that has an antiviral effect against herpes simplex virus types 1 (HSV-1). A study conducted by Pilau et al. (2011) on *Mentha pulegium* L. EOs showed that carvacrol is the main compound and its antiviral effect on humans and animals was investigated. They concluded that these EOs can inhibit different human and animal viruses such as rotavirus, bovine diarrhea virus, and respiratory syncytial virus *in vitro* (Pilau et al., 2011). The mechanism of action and anti-viral activities of carvacrol derived from oregano oil has been explored against murine norovirus (MNV), a non-enveloped virus. Carvacrol was found to help deactivate MNV, a human NoV surrogate, within 1 h of exposure, directly affecting the viral capsid and thereafter RNA (Gilling et al., 2014). Carvacrol inhibits MNV binding to host cells via hiding the capsid, however, there was no altered structural morphology of the virus reported (Gilling et al., 2014).

Carvacrol is a relatively safe agent of plant origin and classified as generally regarded as safe. Even though several antimicrobial agents are not preferred for safety reasons, in this situation, carvacrol would be a good choice as antimicrobials. For example, the application of antimicrobials on foods or food contact surfaces, or it could be used in place of corrosive compounds on surfaces. Carvacrol, isolated from oregano oil, has shown to possess strong antimicrobial activity against multiple pathogenic bacterial species (Ultee et al., 2002; Knowles et al., 2005; Cox and Markham, 2007; Ravishankar et al., 2009; Garcia-Garcia et al., 2011; Mild et al., 2011) and could be beneficial for possible use as a surface (fomite) sanitizer and natural food to manage norovirus (NoV). The mechanism of action of carvacrol on bacteria is quite different due to the complex components and structure of the bacterial cell wall. Further, there are also reports available that show a direct action of carvacrol on the bacterial membrane and cell wall (Ultee et al., 2002; Cox and Markham, 2007; Garcia-Garcia et al., 2011).

## The Safety Profile of Carvacrol

There are numerous beneficial effects of EOs such as antioxidant, antimicrobial, and antimutagenic effects. However, apart from this pharmacological efficacy, EOs may have possible toxicity, for example, genotoxicity/mutagenicity (Llana-Ruiz-Cabello et al., 2015). Carvacrol has been used for a long time in diets as well as for medicinal purposes to support health and well-being. It is one of the vital constituents of numerous herbal formulations. It has been demonstrated that a high concentration of carvacrol (460 μM) may have mutagenicity and genotoxicity effects on the intestinal cell line Caco-2, as it caused DNA damage (Llana-Ruiz-Cabello et al., 2015). However, there were no adverse effects reported in human lymphocytes and hepatocytes as well as the lung fibroblast of Chinese hamsters (LLana-Ruiz-Cabello et al., 2014; Maisanaba et al., 2015).

## Pharmacokinetics of Carvacrol

Carvacrol possesses favorable pharmacokinetic and physicochemical properties to be developed as a drug, based on the popular rules including Lipinski’s, Veber’s, and Egan’s (Egan et al., 2000; Lipinski et al., 2001; Veber et al., 2002) depict drug-like properties. In the intestine, carvacrol is gradually absorbed following oral administration (1.5 g) in rabbits. However, 30% remain in the GI tract and 25% excreted by urine after 22 h (Suntres et al., 2015).

In another study, multiple doses of sesame oil derived carvacrol were administered orally into rabbits (1,500 and 5,000 mg) and rats (500 mg). The results showed its distribution in the intestines, stomach, and urine, with little quantity in the muscle, liver, and lung. The intestinal delivery of carvacrol in animals was significantly enhanced by alginate–whey protein microcapsules with a diameter in the range of 250 and 800 μm, which contain 72 and 76 g/kg of carvacrol, respectively (Wang et al., 2009). Uncapsulated carvacrol has been shown to absorb or metabolize over 95% in duodenum and stomach. Even though microcapsules completely freed the compound in the intestinal tract, a better recovery has been observed in the small intestine than large intestine with larger microcapsules in particular (Wang et al., 2009). It was found that uncapsulated carvacrol greatly absorbed/metabolized in the upper GI tract of pigs upon oral ingestion, while alginate–microcapsules were found to reduce the absorption of carvacrol in the stomach and proximal intestine and enhanced the percentage of carvacrol reached to the distal small intestine (Zhang et al., 2016).

According to Austgulen et al. (1987), the metabolism of carvacrol occurs following two types of pathways. The primary metabolic pathway is the conjugation of the phenolic group with glucuronic acid (C_6_H_1__0_O_7_) and sulfate (SO_4_^2–^), when administered at a low dose, the metabolism of carvacrol covers the terminal methyl groups’ oxidation to primary alcohols (Austgulen et al., 1987). Carvacrol (1 mmol/kg) administration to albino rats showed its excretion in urine in their original form or the form of glucuronide and sulfate conjugates (Austgulen et al., 1987). Dong et al. (2012) revealed the cytochrome P450 role in carvacrol and its isomer thymol metabolism, in microsomes of the human liver. The isoform, CYP2A6 was observed in a primary drug-metabolizing enzyme and generated metabolites following oxidation of carvacrol (Dong et al., 2012).

## Limitations

Among numerous compounds screened to date using *in silico* tools, carvacrol appears to target both, the viral protein as well as the viral entry factors in humans. Carvacrol has also been shown to possess favorable physicochemical properties and appears to be a druggable compound. There is a paucity of preclinical data on the potential of carvacrol on infection, inflammation, and immunity in the context of COVID-19. Thus, the therapeutic efficacy of carvacrol must be tested in currently available preclinical animal models for SARS-CoV-2 infection (Cleary et al., 2020; Munoz-Fontela et al., 2020; Yuan et al., 2020), to explore whether the candidate compounds can be used as a preventive agent or therapeutic adjuvant. To conclude the use in COVID-19, it should be investigated and validated in the preclinical models of COVID-19, despite strong evidence for their anti-inflammatory, immunomodulatory, and antiviral properties in other disease models. However, based on the potent immunomodulatory, anti-inflammatory, and antimicrobial properties and additional *in silico* observations, carvacrol seem to be a suitable candidate for further investigation.

## Conclusion

In summary, based on the wide range of experimental studies, carvacrol, and its metabolites appear to exert protective effects against inflammation, immune dysfunction, and infection within the scope of COVID-19. The strong anti-inflammatory properties mediating multiple mechanisms such as the reduction in pro-inflammatory cytokines, chemokines, and adhesion molecules along with inhibition of macrophage infiltration, neutrophil-endothelial cell interaction, provide a plausible reason to inhibit the cytokine storm, a major player of severity, complications, and death in COVID-19.

Carvacrol as a potent antioxidant and immunomodulator may enhance the host cellular immunity against infections. Carvacrol’s capability in interfering with ACE2 receptors in the host, along with its antiviral properties due to its interaction with viral protease and antibacterial properties further strengthen the candidature of carvacrol in viremia and secondary infections, which eventually lead to complications and fatal outcomes. This review highlight the pharmacological principles and observations outlined in published studies, which plausibly suggest possible anti-inflammatory, antiviral, and immunomodulatory properties in context to COVID-19.

When taken into account, the numerous properties of carvacrol such as immunomodulatory, anti-inflammatory, and antiviral effects, along with its molecular mechanisms, indicate that it could be an important therapeutic candidate for COVID-19. In addition, the pharmacological actions, negligible toxicity, drug likeliness properties of carvacrol indicate that it could be used as a nutraceutical or pharmacological agent and/or adjuvant against COVID-19. However, the use of carvacrol in COVID-19 remains inconclusive until preclinical and clinical data is available on its efficacy and safety and does not advice the use of carvacrol in any forms for COVID-19.

## Author Contributions

HJ drafted the manuscript and performed the correction. MFNM drew the scheme, edited the manuscript, and performed the literature survey. NKJ wrote the manuscript, and edited and improved the scheme artwork. SO conceptualized, wrote, and edited the manuscript, and performed the literature survey and ideation of scheme. All authors contributed to the article and approved the submitted version.

## Conflict of Interest

The authors declare that the research was conducted in the absence of any commercial or financial relationships that could be construed as a potential conflict of interest.
